# Spatial patterns of Anchoveta (*Engraulis ringens*) eggs and larvae in relation to *p*CO_2_ in the Peruvian upwelling system

**DOI:** 10.1098/rspb.2017.0509

**Published:** 2017-05-24

**Authors:** Sara G. Shen, Andrew R. Thompson, Jonathan Correa, Peer Fietzek, Patricia Ayón, David M. Checkley

**Affiliations:** 1 Scripps Institution of Oceanography, University of California San Diego, 9500 Gilman Drive, La Jolla, CA 92093-0208, USA; 2 NOAA Fisheries Service, Southwest Fisheries Science Center, 8901 La Jolla Shores Drive, La Jolla, CA 92037-1508, USA; 3 Instituto del Mar del Perú, Esquina Gamarra y Gral. Valle s/n, Apartado 22, Callao, Lima, Peru; 4 GEOMAR Helmholtz Centre for Ocean Research Kiel, and Kongsberg Maritime Contros GmbH, Wischhofstraße 1-3, 24148 Kiel, Germany

**Keywords:** ocean acidification, Peru, Anchoveta (*Engraulis ringens*), spawning habitat, larvae

## Abstract

Large and productive fisheries occur in regions experiencing or projected to experience ocean acidification. Anchoveta (*Engraulis ringens*) constitute the world's largest single-species fishery and live in one of the ocean's highest *p*CO_2_ regions. We investigated the relationship of the distribution and abundance of Anchoveta eggs and larvae to natural gradients in *p*CO_2_ in the Peruvian upwelling system. Eggs and larvae, zooplankton, and data on temperature, salinity, chlorophyll *a* and *p*CO_2_ were collected during a cruise off Peru in 2013. *p*CO_2_ ranged from 167–1392 µatm and explained variability in egg presence, an index of spawning habitat. Zooplankton abundance explained variability in the abundance of small larvae. Within the main spawning and larva habitats (6–10°S), eggs were found in cool, low-salinity, and both extremely low (less than 200 µatm) and high (more than 900 µatm) *p*CO_2_ waters, and larvae were collected in warmer, higher salinity, and moderate (400–600 µatm) *p*CO_2_ waters. Our data support the hypothesis that Anchoveta preferentially spawned at high *p*CO_2_ and these eggs had lower survival. Enhanced understanding of the influence of *p*CO_2_ on Anchoveta spawning and larva mortality, together with *p*CO_2_ measurements, may enable predictions of ocean acidification effects on Anchoveta and inform adaptive fisheries management.

## 1. Introduction

Ocean acidification is the increase in the partial pressure of CO_2_ (*p*CO_2_) and decrease in pH and CaCO_3_ saturation state caused by the rapid addition of atmospheric CO_2_ to the ocean from deforestation and fossil fuel burning [1]. While declining CaCO_3_ saturation state may make it more difficult for calcifying organisms to secrete their exoskeletons [1,2], increasing *p*CO_2_ may be particularly challenging for fish [3]. Elevated *p*CO_2_ will reduce the outward partial pressure gradient of CO_2_ across the gills and skin of fish, and can lead to respiratory acidosis [3,4]. Many of the reported effects of elevated *p*CO_2_ on fish are assumed to be the downstream consequence of compensatory processes to restore internal pH homeostasis during acidosis.

Several species of fish are susceptible to elevated *p*CO_2_ during the early life-history stages (i.e. eggs and larvae), experiencing changes to physiology, development, growth, behaviour, central neural processing and mortality (see review by [5]). Some of these changes have the potential to reduce fitness and chance of survival. Importantly, high and variable mortality during the larval stage, often driven by adverse environmental conditions, greatly influences recruitment variability [6]. Given the susceptibility of young fish to ocean acidification and their role in shaping fisheries, it is essential to understand the impacts of ocean acidification on this important life stage. Naturally high-*p*CO_2_ areas, such as eastern boundary upwelling systems (EBUS) and CO_2_ vents, offer the opportunity to investigate the current relationship between organisms and the CO_2_ of their environment and pose questions about future changes with ocean acidification [7–9].

Anchovies (*Engraulis* spp.) are small pelagic fish that occur worldwide in temperate regions of high productivity, particularly in the coastal upwelling areas of EBUS [10,11]. Wind-driven upwelling brings cold, nutrient-rich, high-*p*CO_2_ waters to the surface and creates a spatial and temporal mosaic in *p*CO_2_ [7,12]. Anchovy populations around the globe undergo large fluctuations in biomass in response to environmental changes on interannual to centennial timescales [9,13–15]. Notable examples are the collapse of the Pacific sardine (*Sardinops sagax*) fishery off California in 1947 and Anchoveta (*Engraulis ringens*) fishery off Peru in 1972 due to changing ocean conditions and overfishing [11,14]. The low level of nucleotide diversity and shallow coalescence of mitochondrial DNA genealogies of anchovies indicate periodic regional population collapses have occurred in the past in response to changes in oceanographic processes [10]. Rapid evolutionary adaptation is more likely to occur in populations with high levels of existing genetic variation and large population size [7]. Therefore, the strong influence of the environment on the biomass and recruitment combined with the genetic structure suggest that anchovy populations may be especially vulnerable to climate change effects [9].

Anchoveta (*Engraulis ringens*) inhabit the Humboldt Current System and play an important ecological role as a midtrophic-level species [16], and support the world's largest single-species fishery [17]. Of the three stocks, the north-central Peru (NCP, 4–15 °S) stock is located within the highly productive and high-*p*CO_2_ Peruvian upwelling system and dominates the landings [14]. The Peruvian upwelling system experiences elevated *p*CO_2_ year-round, with concentrations exceeding those of other EBUSs. Measurements of *p*CO_2_ in the coastal region can reach 1500 µatm [18–20]. Coastal upwelling systems have a lowered buffering capacity to offset acidification and are at the forefront of observable climate change [18,21]. ‘Hotspots’ of acidification (*p*CO_2_ > 1000 µatm) are predicted to occur in major fishery zones by mid-century when atmospheric CO_2_ is projected to reach 650 µatm [21].

Anchoveta respond to environmental fluctuations by altering their habitat use and reproductive strategy. For example, during El Niño events, Anchoveta migrate further south and nearer to the coast to seek refuge from warm temperatures [13]. Fecundity and spawning frequency are reduced, and the spawning season extended [13,22]. Unlike adults, the early life-history stages of many fish species are planktonic and largely unable to make behavioural modifications to escape stressful environmental conditions. Furthermore, the peak spawning season for Anchoveta (August–November) occurs during maximum upwelling activity [19,23], resulting in the spawning and development of eggs and larvae at high *p*CO_2_.

We use the Peruvian upwelling system, with its naturally high *p*CO_2_ and large Anchoveta population, as a natural experiment to investigate the relationship of pelagic fish eggs and larvae to *p*CO_2_. We posed the following questions: (i) What is the surface water *p*CO_2_ in the spawning habitat? (ii) Are eggs and larvae found in areas of high *p*CO_2_? (iii) Can we use differences in spawning and larva habitats to make inferences about the effects of *p*CO_2_ on mortality? To address these questions, we examined the distribution and abundance of Anchoveta eggs and larvae across an inshore–offshore gradient of *p*CO_2_ off Peru during the spawning and upwelling season in 2013.

## 2. Material and methods

### (a) Cruise information

**Figure 1.**
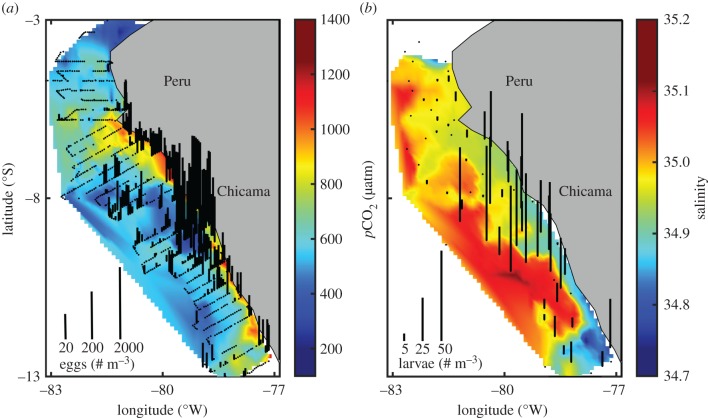
Maps of the concentration of (*a*) eggs and (*b*) larvae of standard length less than 5 mm. Lengths of the black lines correspond to the concentration of eggs and larvae at stations where samples were collected. Interpolated measurements of (*a*) *p*CO_2_ and (*b*) salinity are shown in the background.

Eggs and larvae of the NCP stock of Anchoveta and oceanographic data were collected between 3 °S and 12 °S during a 30 day cruise in August–September 2013. The cruise was conducted by the Instituto del Mar del Perú (Imarpe) and comprised parallel transects extending from the coast to approximately 90 nm (140 km) offshore (figure 1).

### (b) Oceanographic data

Temperature, salinity and *p*CO_2_ were measured continuously and recorded at 1 minute intervals from the vessel's seawater system at 3 m depth using a thermosalinograph (Sea-Bird Electronics Inc., Bellevue, WA, Model SBE-45) and flow-through sensor based on membrane equilibration and non-dispersive infrared spectrometry (KM Contros GmbH, Kiel, Germany, Model HydroC^®^ CO_2_ FT), respectively. Three seawater samples were taken for the analysis of total alkalinity and dissolved inorganic carbon (Andrew Dickson, Scripps Institution of Oceanography, La Jolla, CA, USA) to validate flow-through *p*CO_2_ measurements. An 8 day composite of surface chlorophyll *a* (mg m^−3^) within a 15 km radius of egg and larva stations was obtained from the MODIS Aqua Ocean Color sensor (http://coastwatch.pfeg.noaa.gov). Chlorophyll *a* is a proxy of primary productivity and, in this study, an indication of the availability of phytoplankton prey for young larvae since Anchoveta larva prey on phytoplankton until 2.5–4.0 mm in standard length (SL) [24,25].

### (c) Eggs and larvae

Eggs were collected continuously at a depth of 3 m in 20 minute intervals using the Continuous Underway Fish Egg Sampler (CUFES), which concentrates and filters eggs from a flow of seawater through 330-μm mesh [26]. Volumetric concentration (eggs min^−1^ and eggs m^−3^) at 3 m is highly correlated with areal abundance (eggs m^−2^) [26,27]. Eggs were counted onboard and preserved in 2% formalin-seawater.

Larvae ranging from 2 to 15 mm were collected on station using a Hensen net of 60 cm diameter and 330 μm mesh towed vertically from a depth of 50 m [28]. Larvae were counted, measured to the nearest 0.1 mm, and preserved in 70% ethanol. Larvae measuring less than 5 mm in length were used for subsequent analyses because they were more abundant than larger larvae and their collection was more contemporaneous with oceanographic measurements. Lengths were not adjusted for shrinkage and counts were not adjusted for variable retention rate as there is evidence for both complete [29,30] and partial (0.63) retention [31] of anchovy larvae smaller than 5 mm caught using 330 μm mesh. Because small larvae (less than 5 mm) can actively swim only approximately 50% of the time and at speeds that are significantly lower than typical current speeds [25,32], their behavioural contribution to horizontal movement is negligible and they are not expected to avoid the net. However, bias in abundance data may be present.

The remaining zooplankton were preserved in 2% formalin-seawater buffered with borax. Zooplankton volume was measured using the displacement method following the removal of large gelatinous organisms [28] and is used here as an index for the abundance of zooplankton that prey on Anchoveta larvae. Maps of the concentration of eggs and larvae with *p*CO_2_ and salinity interpolations to a 0.1° grid were created using MATLAB (The Mathworks, Inc., Natick, MA, USA).

### (d) *p*CO_2_ data processing

The HydroC^®^ CO_2_ FT automatically performed zero-CO_2_ gas measurements every 12 h. Zero-CO_2_ gas readings and pre- and post-calibration information were used to apply a drift correction based on absolute sensor runtime [33]. The response time (RT) of the sensor varied due to a variable flow of the ship's seawater system and fouling. To account for the effect of variable RTs, two corrections were performed based on signal recovery times following zero-CO_2_ gas measurements [34], a ‘slow/fouled’ RT of 1200 s and ‘fast/unfouled’ RT of 300 s. The final RT-corrected series was created from these two datasets. Time periods of variable seawater flow were removed from the final data as their quality was unknown.

A linear regression (*R*^2^ = 0.99) of temperature measurements at 3 m depth obtained from Niskin casts during sampling stations (*n* = 50) and temperature measurements from the thermosalinograph was used to convert the RT-corrected *p*CO_2_ at the sensor to *p*CO_2_ *in situ* at 3 m [35]. *p*CO_2_ measurements from the HydroC^®^ CO_2_ FT and those estimated from DIC and TA measurements using CO_2_Calc (http://pubs.usgs.gov/of/2010/1280/) had an average deviation of 2.2%. We attribute an uncertainty of 1.5% for the majority of measurements, but acknowledge that over short periods and in times of large *p*CO_2_ gradients, the uncertainty could be as high as 10% due to discrepancies between the sensor's actual RT and that assumed during processing.

### (e) Spatial generalized linear mixed models

We constructed a set of candidate models (electronic supplementary material, table S1) to evaluate the relative influence of temperature, salinity, *p*CO_2_, satellite chlorophyll *a* and zooplankton displacement volume on the presence of eggs and abundance of small larvae (SL <5 mm). Egg presence was modelled to capture the areal extent of the spawning habitat and because 60% of CUFES samples contained zero eggs [36,37].

We performed logistic regressions with a binomial distribution and logit link to model the effects of temperature, salinity, *p*CO_2_ and chlorophyll *a* on egg presence. Larva abundance was modelled using generalized linear models with a Poisson distribution and log link. Zooplankton displacement volume was included as an additional predictor variable. Models included either temperature or salinity, and either temperature or *p*CO_2_ due to strong correlations between these variables (Pearson's |*r|* > 0.6; electronic supplementary material, table S2). Quadratic terms were included in models because the probability of encountering eggs of anchovy can peak across a range of oceanographic conditions [36,38] and dome-shaped relationships may be indicative of an ‘optimal environmental window’ [39].

Egg and larvae data were standardized by subtracting the mean and dividing by the standard deviation prior to model fitting. Spatial autocorrelation, detected by global Moran's I, was accounted for as a random effect through the use of spatial generalized linear mixed models (SGLMM) using the function corrHLfit in the package ‘spaMM’ in R v. 3.1.2 (R Core Team 2013) [40].

The relative plausibility of the candidate SGLMMs was determined using Akaike's Information Criterion adjusted for small sample sizes (AICc; electronic supplementary material, table S3) [41]. We calculated the ΔAICc and scaled the models by their Akaike weight. Parameter estimates for each variable in models with Akaike weights greater than 10% of the model with the lowest AIC were averaged to account for model selection uncertainty using the R package ‘AICcmodavg’ [41]. Ninety-five per cent confidence intervals were constructed around parameter estimates for each predictor variable in the model. Parameters were interpreted as significant if confidence intervals did not overlap zero [41].

We constructed partial-effects plots to illustrate the effect of individual predictor variables on the probability of egg capture and predicted number of larvae. Partial effects were calculated by allowing the variable of interest to take on measured values while all other predictor variables in the model were fixed at their median value [37]. Predicted probabilities and counts were averaged within 0.5 unit bins to provide a clearer picture of the central relationships.

## 3. Results and discussion

### (a) *p*CO_2_

*p*CO_2_ ranged from 167–1392 µatm, consistent with the range of approximately 150–1500 µatm measured during 2004–2006 in this region [19]. In general, *p*CO_2_ was high near to the coast where wind-driven coastal upwelling occurred and decreased offshore. Approximately 74% of the measurements during 2004–2006 exceeded atmospheric *p*CO_2_ (378 µatm; http://www.esrl.noaa.gov/gmd/ccgg/globalview/index.html) by more than 100 µatm and 8% had values that were more than twice atmospheric *p*CO_2_ [19]. In comparison, 84% of our data exceeded atmospheric *p*CO_2_ (393 µatm) by more than 100 µatm and 23% were greater than twice atmospheric *p*CO_2_ (756 µatm). However, the majority of high values (*p*CO_2_ > 1000 µatm) in 2004–2006 were observed further south (14–16 °S) than the main spawning area and our study region. Our findings indicate that the spatial extent of high *p*CO_2_ water in the main spawning habitat, as well as the maximal concentration of *p*CO_2_ in this water, was greater in 2013 compared with 7–9 years ago.

### (b) Spawning habitat characterization

Eggs were found throughout the sampling region in high abundance. A total of 236 220 eggs were collected in 867 CUFES samples, with a mean of 27 eggs m^−3^ and maximum of 2000 eggs m^−3^ (figure 1*a*). Maximal egg concentration was 1–3 orders of magnitude greater than that of the central-south Chile stock of *E. ringens* (33–42°S) [14,42], *E. mordax* in the California Current [26,36] and *E. encrasicolus* in the Benguela Current [43], and reflects the large spawning stock biomass off Peru.

**Figure 2.**
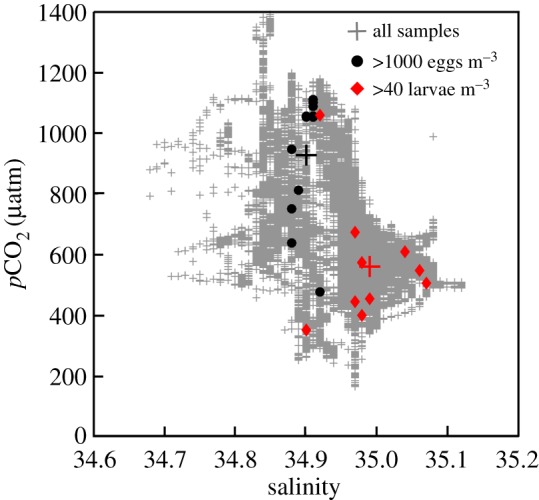
*p*CO_2_-salinity diagram for all seawater measurements (grey plus signs), CUFES samples containing more than 1000 eggs m^−3^ (black circles) and samples of larvae containing more than 40 larvae m^−3^ (red diamonds). The centroids of the distributions are indicated by the black and red plus signs.

The main spawning habitat was located between 7–10°S (figure 1*a*), consistent with other years [23,44–46]. This area is characterized by a wider continental shelf, increased stability of the physical environment, higher retention rates, and better feeding conditions for larvae, all factors that likely contribute to making it a preferred spawning location [23,32,45]. CUFES samples with the highest concentrations of eggs (more than 1000 eggs m^−3^) were found near to the coast in recently upwelled water with a mean salinity and *p*CO_2_ of 34.90 ± 0.01 (mean ± s.d.) and 930 ± 211 µatm, respectively (figures 1*a* and 2). Spawning also occurred offshore in lower *p*CO_2_ water (figure 1*a*).

**Figure 3.**
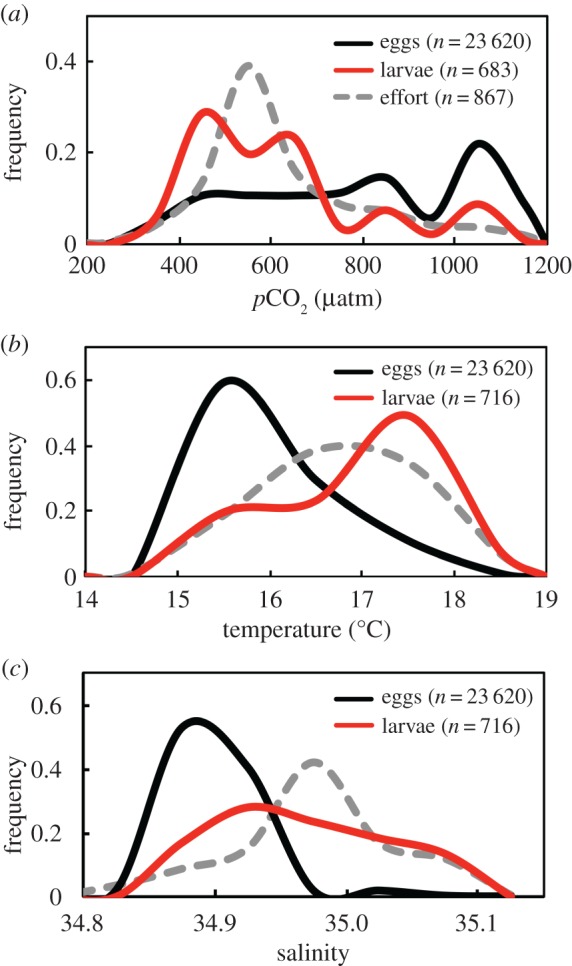
Frequency distributions of eggs (black lines), larvae (red lines) and sampling effort (dashed grey lines) in relation to (*a*) *p*CO_2_, (*b*) temperature and (*c*) salinity. Data for *p*CO_2_, temperature and salinity were binned into 100-μatm, 0.5°C and 0.05-intervals and a spline was performed to generate smooth curves.

Egg frequency of occurrence was maximal in water of high *p*CO_2_ (1000–1100 µatm), cold temperature (15–16°C) and relatively low salinity (34.85–34.90) (figure 3). Chlorophyll *a* estimated from satellite imagery ranged from 0.2 to 16.7 mg m^−3^ and eggs occurred most frequently in areas with high chlorophyll *a* concentrations. These oceanographic conditions are characteristic of the productive cold coastal water mass (CCW) that dominates coastal upwelling [47].

**Figure 4.**
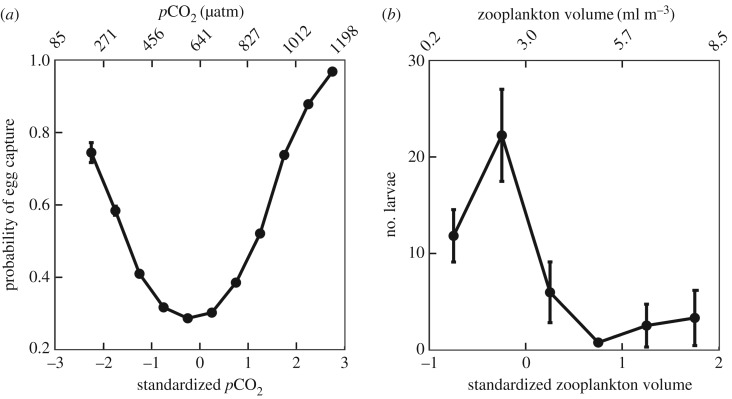
Partial effects diagrams of (*a*) *p*CO_2_ on the probability of egg capture and (*b*) zooplankton displacement volume on the abundance of larvae. Data were standardized prior to model fitting. Mean and standard error are shown for bins of 0.5 unit.

*p*CO_2_ was the only statistically significant variable to predict egg presence (table 1). The relationship of *p*CO_2_ to egg presence was positive and quadratic (table 1; figure 4*a*). The probability of collecting eggs increased from 0.30 to 0.97 as *p*CO_2_ increased from the mean of 641 µatm to 1198 µatm (figure 4*a*), indicative of the fact that spawning was largely associated with upwelled water high in *p*CO_2_. The high probability of finding eggs in the lowest *p*CO_2_ waters was initially unexpected, but corroborates the offshore spawning that was observed in low *p*CO_2_ water (figure 1*a*).

**Table 1.** Standardized, model-averaged parameter estimates and lower and upper 95% confidence intervals (LCI, UCI) for candidate models that describe the relationship between egg presence and abundance of larvae, and oceanographic variables. Quadratic terms are denoted as the parameter squared. Data were standardized prior to model fitting by subtracting the mean and dividing by the standard deviation. Significant (*p*-value < 0.05) parameter estimates are in italics.

variable	eggs			larvae		
	estimate	LCI	UCI	estimate	LCI	UCI
Temp	−0.12	−0.30	0.06	0.10	−0.30	0.49
Temp^2^	0.14	0.00	0.28	−0.02	−0.19	0.15
Sal	−0.06	−0.25	0.13	0.02	−0.15	0.19
Sal^2^	0.02	−0.10	0.14	0.00	−0.11	0.11
pCO_2_	0.19	−0.01	0.38	−0.03	−0.24	0.18
*p*CO_2_^2^	*0.52*	*0.37*	*0.68*	0.02	−0.13	0.17
Chl	0.02	−0.30	0.34	0.04	−0.33	0.41
Chl^2^	−0.02	−0.10	0.06	−0.02	−0.16	0.13
Zoo	—	—	—	0.21	−0.44	0.85
Zoo^2^	—	—	—	*−0.37*	*−0.73*	*−0.01*

To our knowledge, this is the first report of *p*CO_2_ as a variable that significantly characterizes the spawning habitat of an anchovy species. Temperature and salinity, often with chlorophyll *a* concentration, have been identified as important factors in the characterization of spawning habitat for anchovy in the California Current [27,36,37,48] and Benguela Current [43]. A strong, positive relationship between egg abundance and prey availability was found for Anchoveta off Chile [42]. Our results are consistent with others [48,49] showing temperature and salinity alone do not define the spawning habitat of Anchoveta in the Humboldt Current.

### (c) Larva habitat characterization

A total of 1157 Anchoveta larvae were collected at 74 stations, of which 683 measured less than 5 mm in SL and had corresponding *p*CO_2_ data for subsequent analyses. Larvae were concentrated between 7 and 10°S, and samples had a mean of 10 larvae m^−3^ and a maximum of 51 larvae m^−3^ (figure 1*b*).

Compared with eggs, larvae were more abundant in lower *p*CO_2_ (400–500 µatm), warmer (17–18°C), and more saline (34.90–35.00) water (figure 3). Selection for *p*CO_2_ was not evident, with 48% of larvae found between 400–600 µatm, corresponding to the *p*CO_2_ range most sampled (figure 3). Chlorophyll *a* concentration ranged from 0.3 to 5.8 mg m^−3^ and zooplankton displacement volume ranged from 0.2 to 7.5 mg m^−3^.

Larvae were found within the CCW, but to a larger extent within the mixed coastal-subtropical water mass (MCS), characterized by higher temperatures and salinity [42]. Only 17% of larvae were found at salinity <34.90, where the majority of eggs were collected. Mean salinity and *p*CO_2_ were 34.98 ± 0.06 and 552 ± 183 µatm, respectively, for the largest samples containing more than 40 larvae m^−3^ (figure 2).

**Figure 5.**
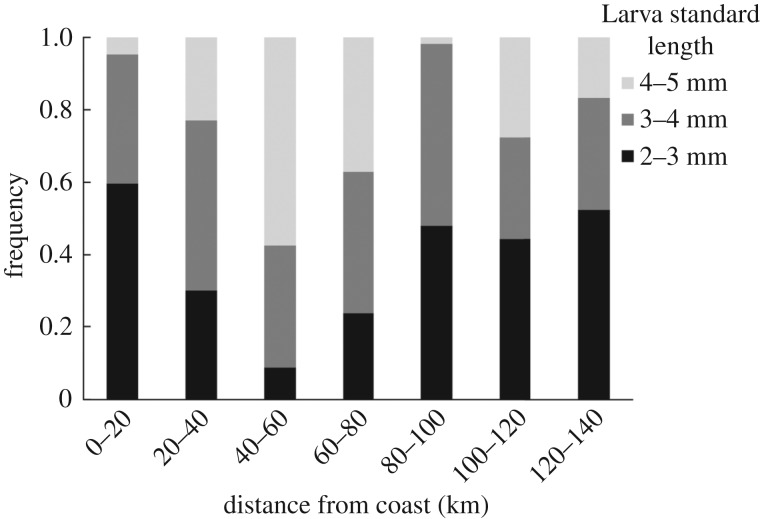
Stacked frequency histograms of larvae with respect to distance from the coast. Data are for larvae with standard length of 2–3 mm (black bars), 3–4 mm (dark grey bars) and 4–5 mm (light grey bars) in distance bins of 20 km from the coast (0 km) to the furthest stations offshore (140 km).

Larvae of various lengths (ages) were widespread throughout the larva habitat (figure 5). As an example, 52% of the larvae that were collected between 120 and 140 km were between 2–3 mm, 31% between 3–4 mm and 17% between 4–5 mm. Similarly, large larvae were also found near shore, with 23% of the larvae between 3–4 mm at stations 20–40 km from the coastline (figure 5).

Zooplankton displacement volume significantly explained larva abundance (table 1). The relationship was negative and quadratic (table 1; figure 4*b*). Larva abundance increased as zooplankton volume reached the mean value of 3.0 ml m^−3^ and then decreased exponentially as volume increased (figure 4*b*). Because zooplankton were collected using a 330 μm mesh, zooplankton volume provides an estimate of the abundance of zooplankton predators of larvae. Anchoveta larvae transition from a diet of phytoplankton to zooplankton around 4.3 mm, but the main prey, copepod nauplii (40–90 µm in diameter) [25], are too small to have been captured in zooplankton collections. Therefore, the decrease in larva abundance at high zooplankton volume is consistent with higher predation rates by zooplankton predators on larvae. Similarly, the occurrence of Anchoveta larvae off Chile was negatively correlated with predatory zooplankton [27] and positively correlated with small microplankton [42].

### (d) Factors influencing habitat characterizations

The spawning and larva habitats of the NCP stock of Anchoveta were similar in latitudinal range, but distinct in oceanographic conditions. Samples with the highest concentrations of eggs and larvae were collected in waters of significantly different salinity and *p*CO_2_ (*t*-test, *p*-value < 0.001), corresponding to different water masses. Egg frequency of occurrence, probability of collection and concentration were maximal at high *p*CO_2_ and low salinity in CCW. Contrarily, larva frequency of occurrence and concentration were highest at low *p*CO_2_ and high salinity in MCS. There are several factors to consider that could explain the distinct habitat characterizations for eggs and larvae.

Although advection has the potential to transport individuals offshore, natal locations [50], oceanographic modelling and our data indicate that offshore advection is minimal in our study area. Regional hydrodynamic and individual-based models, which account for egg buoyancy and larval vertical migration, concluded that retention is upward of 70% in the main spawning and larva habitats (7–10 °S) [23,51]. Over an 8 day period drifting at 4 cm s^−1^, eggs and larvae are transported less than 28 km in simulations [23]. In addition, the relative strength of alongshore currents in the winter would support equatorward rather than offshore transport [51]. Lastly, direct observation of our own data also does not support significant offshore transport during development. Both eggs and larvae of various lengths were found throughout the sampling region, from near-shore stations to those further offshore (figures 1 and 5). Indeed, it is thought that spawning in locations that favour retention is a fundamental life-history characteristic of Anchoveta in the Humboldt Current [51,52].

In general, *p*CO_2_ is high along the coast where waters are upwelled and decreases offshore [18,19,53]. As upwelled water ages, *p*CO_2_ declines primarily due to the uptake of inorganic carbon by phytoplankton for primary production [19,53]. Drifters in the Peruvian and Mauritanian upwelling systems measured an average decline in *p*CO_2_ of less than 200 µatm over an approximately one-week period of offshore transport [19,53]. We collected and analysed data of eggs, which hatch in approximately 2 days at 17°C [44], and larvae of SL <5 mm, corresponding to first-feeding larvae approximately one week in age [25]. Despite variability in *p*CO_2_ on the event time scale from changes in wind, upwelling and primary production, we anticipate a decline in *p*CO_2_ of 200 µatm during the 5–7 day development of eggs into young larvae.

We hypothesize that the differences we observed in the distributions of eggs and larvae result from low egg and larva survival at high *p*CO_2_. Our results suggest that eggs spawned at high *p*CO_2_ (more than 900 µatm) suffered higher mortality than those spawned at lower *p*CO_2_, contributing to the absence of large concentrations of larvae at *p*CO_2_ > 700 µatm. Despite the majority of spawning having occurred at high *p*CO_2_, a larger fraction of eggs spawned further offshore in waters of higher salinity and low-to-intermediate *p*CO_2_ appear to have survived to the larval stage. Although this study was not specifically designed to measure mortality, observations of distinct egg and larva habitats, coupled with the significance of *p*CO_2_ as the variable characterizing spawning habitat in SGLMMs, are consistent with the hypothesis that the concentration of larvae relative to eggs in different water masses is representative of regional differences in mortality rate associated with *p*CO_2_.

We acknowledge that there are variables that we did not measure that may have affected the distribution and abundance of eggs and larvae, and whose mechanistic relationship may have been captured by the environmental variables we measured. Oxygen is strongly correlated with *p*CO_2_ [54] and an important variable affecting fish distributions in the Humboldt Current [55]. However, while the correlation of O_2_ and *p*CO_2_ is strong at depth, O_2_ in the surface ocean is near saturation despite elevated *p*CO_2_. Of the 47 samples of surface (0 m depth) O_2_ taken during the cruise, 15 of these were taken in seawater with *p*CO_2_ > 800 µatm and O_2_ ranged from 2.15 to 5.18 ml l^−1^. Additionally, variables related to water column structure and horizontal flow have increased the power of models to predict anchovy spawning habitat in the California Current [36,37]. Lastly, the inshore–offshore gradient in upwelling that drives the spatial pattern in *p*CO_2_, O_2_ and horizontal flow, also influences nutrient concentrations and the community composition and size structure of phytoplankton and zooplankton. All of these factors are likely to affect the distribution, abundance and survival of eggs and young larvae. Therefore, measurements of these variables in addition to *p*CO_2_ are needed during future cruises to rigorously test our hypothesis that *p*CO_2_ is an important driver of mortality.

### (e) Implications

Using systems such as the Peruvian upwelling that are naturally high in *p*CO_2_ as a window into a future acidified ocean can add value to laboratory experiments. By exploring the spatial patterns of Anchoveta eggs and larvae to *p*CO_2_ in their natural habitat, the entirety of the whole ecosystem, including predator–prey dynamics and other important environmental variables, is taken into consideration. While controlled laboratory experiments may not show a significant effect of elevated *p*CO_2_ on the survival of eggs of Atlantic herring (*Clupea harengus*), mortality in nature might increase due to a decrease in growth rate that would prolong exposure of vulnerable larvae to predators [56]. Similarly, the lack of effect of elevated *p*CO_2_ on Baltic cod (*Gadus morhua*) egg and larva survival in laboratory experiments could be attributed to adaptation since spawning occurs at high *p*CO_2_ [57]. However, the capacity for a species to adapt may be influenced by the source (i.e. upwelling, eutrophication) and history of high *p*CO_2_ in their natural habitat.

Initially, our results appear to contradict the fact that anchovy across EBUSs thrive during La Niña years, characterized by strong upwelling and presumably high-*p*CO_2_ waters. Upon closer examination, our findings do not conflict with this observation and highlight the important role of spatial and temporal scale. For example, our data do not indicate that the Anchoveta population will be reduced during high-*p*CO_2_ years (La Niña events). Rather, our data show that the survivors into the larva stage may originate from eggs spawned further offshore in lower *p*CO_2_ waters during years of strong upwelling with high *p*CO_2_ inshore. In fact, there is precedent for differences in survival with respect to spatial scale, with survivors to six months of age originating from eggs spawned less than 75 km and also more than 150 km offshore in the years 1999, 2003, 2005 and 2007 [58].

As the extent and concentration of high-*p*CO_2_ water increases, ocean acidification has the potential to influence variability in mortality on seasonal and inter-annual time scales. The peak spawning season for Anchoveta is from August–November, when upwelling and abundance of high-*p*CO_2_ water are at a maximum [19,23]. During 1953–1981, recruitment into the fishery at six months was on average larger for the smaller spawning season of February–March than the peak spawning season from August–November [59]. While it was hypothesized that differences in recruitment success could be related to the effects of turbulence on larval food concentration [59], the influence of high-*p*CO_2_ water on mortality could become a contributing factor in the near future. Changes in mortality on spatial and temporal scales could have widespread effects on the population and fishery.

Anchoveta, Alaska pollock (*Theragra chalcogramma*), Skipjack tuna (*Katsuwonus pelamis*), sardines (*Sardinops* spp.) and Atlantic herring (*Clupea harengus*) collectively comprise 19% of the world's global marine fish catch [17]. The major fisheries of the world are largely found in naturally high-*p*CO_2_ regions that are expected to experience ocean acidification earlier and more strongly than other areas of the world's ocean [21]. Thus, understanding the effects of ocean acidification on marine fish is important for the management and sustainability of fisheries in the future. Enhanced understanding requires the long-term monitoring of *p*CO_2_ concurrent with the collection of eggs and larvae. Furthermore, time series of observations spanning multiple decades are required in order to differentiate the impacts of climate variability from those of climate change [9].

The management strategies of Anchoveta and Pacific sardine (*Sardinops sagax*) incorporate climate variability to some degree, and may serve as a model for the sustainable management of fisheries in the face of climate change. For example, Imarpe's EUREKA Program enables managers to repurpose the fishing fleet for a rapid stock assessment of Anchoveta during the onset of an El Niño event [60]. The harvest control rule for Pacific sardine (*Sardinops sagax*) depends on temperature in the southern California Current System [61]. These progressive management strategies are testimony to the importance of, and ability to, consider the impacts of climate on the sustainability of commercial fisheries. In the future, EUREKA cruises and harvest control rules may be triggered by extreme upwelling or La Niña events that are characterized by high *p*CO_2_ waters. It is timely to consider how climate change, particularly ocean acidification, may be incorporated into fishery management strategies.

## Supplementary Material

Supplementary Tables

## Supplementary Material

Supplementary Datasets

## Data accessibility

The datasets supporting this article have been uploaded as part of the electronic supplementary material.
