# Supplementary material for: Spatial patterns of Anchoveta (Engraulis ringens) eggs and larvae in relation to pCO2 in the Peruvian upwelling system
Source: Proc Biol Sci. 2017 May 24;284(1855):20170509. doi: 10.1098/rspb.2017.0509 (PMC5454269; doi:10.1098/rspb.2017.0509)
Supplement: Supplementary Tables [file rspb20170509supp1.docx]

**Spatial patterns of Anchoveta (*Engraulis ringens*) eggs and larvae in relation to *p*CO_2_ in the Peruvian upwelling system**

Sara G. Shen*^a^, Andrew R. Thompson^b^, Jonathan Correa^c^, Peer Fietzek^d^, Patricia Ayón^c^, and David M. Checkley, Jr.^a^

^a^ Scripps Institution of Oceanography, University of California San Diego, 9500 Gilman Drive, La Jolla, CA 92093-0208, USA; ^b^ NOAA Fisheries Service, Southwest Fisheries Science Center, 8901 La Jolla Shores Drive, La Jolla, CA 92037-1508, USA; ^c^ Instituto del Mar del Perú, Esquina Gamarra y Gral. Valle s/n, Apartado 22, Callao, Lima, Peru; ^d^ GEOMAR Helmholtz Centre for Ocean Research Kiel, and Kongsberg Maritime Contros GmbH, Wischhofstraße 1-3, 24148 Kiel, Germany

*To whom correspondence should be addressed: Sara Shen

E-mail: saragshen@gmail.com

**SM Table 1**

| Candidate Models | | | |
| --- | --- | --- | --- |
| Eggs |  |  |  |
| Chl + Chl^2^ |  |  |  |
| Temp + Temp^2^ | |  |  |
| pCO_2_ + pCO_2_^2^ | |  |  |
| Sal + Sal^2^ |  |  |  |
| Temp + Temp^2^ + Chl + Chl^2^ | | |  |
| Sal + Sal^2^ + Chl + Chl^2^ | |  |  |
| Sal + Sal^2^ + pCO_2_ + pCO_2_^2^ | | |  |
| Chl + Chl^2^ + pCO_2_ + pCO_2_^2^ | | |  |
| Sal + Sal^2^ + pCO_2_ + pCO_2_^2^ + Chl + Chl^2^ | | | |
|  |  |  |  |
| Larvae | | | |
| Chl + Chl^2^ |  |  |  |
| Temp + Temp^2^ | |  |  |
| pCO_2_ + pCO_2_^2^ | |  |  |
| Sal + Sal^2^ |  |  |  |
| Zoo + Zoo^2^ |  |  |  |
| Temp + Temp^2^ + Chl + Chl^2^ | | |  |
| Temp + Temp^2^ + Zoo + Zoo^2^ | | |  |
| Sal + Sal^2^ + Chl + Chl^2^ | |  |  |
| Sal + Sal^2^ + pCO_2_ + pCO_2_^2^ | | |  |
| Sal + Sal^2^ + Zoo + Zoo^2^ | |  |  |
| pCO_2_ + pCO_2_^2^ + Zoo + Zoo^2^ | | |  |
| Chl + Chl^2^ + pCO_2_ + pCO_2_^2^ |  |  |  |
| Chl + Chl^2^ + Zoo + Zoo^2^ | |  |  |
| Temp + Temp^2^ + Chl + Chl^2^ + Zoo + Zoo^2^ | | | |
| Sal + Sal^2^ + pCO_2_ + pCO_2_^2^ + Chl + Chl^2^ | | | |
| Sal + Sal^2^ + pCO_2_ + pCO_2_^2^ + Zoo + Zoo^2^ | | | |
| Sal + Sal^2^ + Chl + Chl^2^ + Zoo + Zoo^2^ | | |  |
| pCO_2_ + pCO_2_^2^ + Chl + Chl^2^ + Zoo + Zoo^2^ | | | |
| Sal + Sal^2^ + pCO_2_ + pCO_2_^2^ + Chl + Chl^2^ + Zoo + Zoo^2^ | | | |

Candidate models that describe the relationship between egg presence and abundance of larvae, and oceanographic variables. Quadratic terms are denoted as the parameter squared. Data on zooplankton displacement volume were only available for larvae and therefore zooplankton displacement volume (Zoo) is not included in candidate models for egg presence. Models included temperature or salinity, and temperature or *p*CO_2_ due to strong correlations between these variables (Pearson’s |*r|* > 0.6; SM Table 2).

**SM Table 2**

|  | Eggs | | | Larvae | | | |
| --- | --- | --- | --- | --- | --- | --- | --- |
| Variable | Temp | pCO_2_ | Chl | Temp | pCO_2_ | Chl | Zoo |
| Sal | 0.77 | -0.45 | -0.51 | 0.75 | -0.36 | -0.49 | 0.03 |
| Temp |  | -0.63 | -0.43 |  | -0.69 | -0.46 | -0.14 |
| pCO_2_ |  |  | 0.25 |  |  | 0.26 | -0.02 |
| Chl |  |  |  |  |  |  | -0.01 |

Pearson’s *r* correlations between oceanographic variables in the data sets for eggs (*n* = 724) and larvae (*n* = 65).

**SM Table 3**

| Candidate Models | |  |  |  |  |  |
| --- | --- | --- | --- | --- | --- | --- |
| Eggs |  |  |  | AICc | ΔAICc | Weight |
| pCO_2_ + pCO_2_^2^ | |  |  | 881.5 |  | 0.7 |
| Chl + Chl^2^ + pCO_2_ + pCO_2_^2^ | | |  | 884.9 | 3.4 | 0.1 |
| Sal + Sal^2^ + Chl + Chl^2^ | |  |  | 884.9 | 3.4 | 0.1 |
| Sal + Sal^2^ + pCO_2_ + pCO_2_^2^ | | |  | 952.8 | 71.3 | 0.0 |
| Sal + Sal^2^ |  |  |  | 954.4 | 72.9 | 0.0 |
| Temp + Temp^2^ + Chl + Chl^2^ | | |  | 964.4 | 82.9 | 0.0 |
| Chl + Chl^2^ |  |  |  | 965.2 | 83.8 | 0.0 |
| Temp + Temp^2^ | |  |  | 968.6 | 87.1 | 0.0 |
| Sal + Sal^2^ + pCO_2_ + pCO_2_^2^ + Chl + Chl^2^ | | | | 976.8 | 95.3 | 0.0 |
|  |  |  |  |  |  |  |
| Larvae |  |  |  | AICc | ΔAICc | Weight |
| Zoo + Zoo^2^ |  |  |  | 384.2 | 0.0 | 0.5 |
| Temp + Temp^2^ + Zoo + Zoo^2^ | | |  | 385.6 | 1.4 | 0.2 |
| pCO_2_ + pCO_2_^2^ + Zoo + Zoo^2^ | | |  | 387.2 | 3.0 | 0.1 |
| Sal + Sal^2^ + Zoo + Zoo^2^ | |  |  | 387.9 | 3.7 | 0.1 |
| Chl + Chl^2^ + Zoo + Zoo^2^ | |  |  | 388.2 | 4.1 | 0.1 |
| Temp + Temp^2^ + Chl + Chl^2^ + Zoo + Zoo^2^ | | | | 389.1 | 4.9 | 0.0 |
| Sal + Sal^2^ + pCO_2_ + pCO_2_^2^ + Zoo + Zoo^2^ | | | | 390.6 | 6.4 | 0.0 |
| Sal + Sal^2^ + Chl + Chl^2^ + Zoo + Zoo^2^ | | |  | 391.5 | 7.3 | 0.0 |
| pCO_2_ + pCO_2_^2^ + Chl + Chl^2^ + Zoo + Zoo^2^ | | | | 391.8 | 7.6 | 0.0 |
| Temp + Temp^2^ | |  |  | 392.0 | 7.9 | 0.0 |
| pCO_2_ + pCO_2_^2^ | |  |  | 393.5 | 9.4 | 0.0 |
| Sal + Sal^2^ |  |  |  | 394.0 | 9.8 | 0.0 |
| Chl + Chl^2^ |  |  |  | 394.6 | 10.4 | 0.0 |
| Sal + Sal^2^ + pCO_2_ + pCO_2_^2^ + Chl + Chl^2^ + Zoo + Zoo^2^ | | | | 394.9 | 10.7 | 0.0 |
| Temp + Temp^2^ + Chl + Chl^2^ | | |  | 396.0 | 11.8 | 0.0 |
| Sal + Sal^2^ + pCO_2_ + pCO_2_^2^ | | |  | 396.5 | 12.4 | 0.0 |
| Chl + Chl^2^ + pCO_2_ + pCO_2_^2^ |  |  |  | 398.0 | 13.8 | 0.0 |
| Sal + Sal^2^ + Chl + Chl^2^ | |  |  | 398.0 | 13.9 | 0.0 |
| Sal + Sal^2^ + pCO_2_ + pCO_2_^2^ + Chl + Chl^2^ | | | | 401.1 | 16.9 | 0.0 |

Summary results of model selection for the relationships between egg presence and abundance of larvae, and oceanographic variables. Values are Akaike Information Criterion scores corrected for small sample size (AICc), the change in AICc from the lowest AICc score (ΔAICc), and the Akaike weights for candidate models.
